# When Dendritic Cells Go Viral: The Role of Siglec-1 in Host Defense and Dissemination of Enveloped Viruses

**DOI:** 10.3390/v12010008

**Published:** 2019-12-19

**Authors:** Daniel Perez-Zsolt, Javier Martinez-Picado, Nuria Izquierdo-Useros

**Affiliations:** 1 IrsiCaixa AIDS Research Institute, Ctra. de Canyet s/n, 08916 Badalona, Spain; dperez@irsicaixa.es; 2 Department of Biochemistry and Molecular Biology, Universitat Autònoma de Barcelona, 08193 Bellaterra, Spain; 3 Institut d’Investigació en Ciències de la Salut Germans Trias i Pujol, 08916 Badalona, Spain; 4 Chair in Infectious Diseases and Immunity, Faculty of Medicine, University of Vic-Central University of Catalonia (UVic-UCC), 08500 Vic, Spain; 5 Catalan Institution for Research and Advanced Studies (ICREA), 08010 Barcelona, Spain

**Keywords:** Siglec-1, dendritic cells, HIV-1, Ebola virus, immune evasion

## Abstract

Dendritic cells (DCs) are among the first cells that recognize incoming viruses at the mucosal portals of entry. Initial interaction between DCs and viruses facilitates cell activation and migration to secondary lymphoid tissues, where these antigen presenting cells (APCs) prime specific adaptive immune responses. Some viruses, however, have evolved strategies to subvert the migratory capacity of DCs as a way to disseminate infection systemically. Here we focus on the role of Siglec-1, a sialic acid-binding type I lectin receptor potently upregulated by type I interferons on DCs, that acts as a double edge sword, containing viral replication through the induction of antiviral immunity, but also favoring viral spread within tissues. Such is the case for distant enveloped viruses like human immunodeficiency virus (HIV)-1 or Ebola virus (EBOV), which incorporate sialic acid-containing gangliosides on their viral membrane and are effectively recognized by Siglec-1. Here we review how Siglec-1 is highly induced on the surface of human DCs upon viral infection, the way this impacts different antigen presentation pathways, and how enveloped viruses have evolved to exploit these APC functions as a potent dissemination strategy in different anatomical compartments.

## 1. Introduction

Dendritic cells (DCs) are the most potent antigen presenting cells (APCs) found in humans [1,2] and their immune function is key to initiate immunity against invading viruses [3,4,5]. These cellular sentinels patrol distinct mucosae and upon infection, viral sensing triggers rapid innate immune responses that might initially contain viral spread. DC activation also elicits cellular migration towards secondary lymphoid tissues, where DCs acquire a fully mature phenotype and become competent for antigen presentation, activation of naïve T cells, and expansion of antigen-specific adaptive T cell responses [1,2].

Despite the immune activity exerted by DCs after viral infection, it has been known for decades that viruses evolved different strategies to escape DC antiviral activity [6,7,8]. Furthermore, certain viruses exploit the immune function of DCs as a way to colonize distant tissues and effectively disseminate systemically [9,10,11,12,13,14,15,16,17,18]. The discovery of the role of the receptor Siglec-1/CD169, a sialic acid-binding Ig-like lectin-1 expressed by DCs, has greatly contributed to our understanding of how viruses subvert DC activity. The Siglec-1 receptor acts as an immuno-surveillance molecule [19] but can also be effectively hijacked by distinct enveloped viruses, which either infect DCs directly or are effectively transferred to bystander target cells that become productively infected [20,21,22]. Hence, Siglec-1 function on DCs clearly illustrates how these APCs can trigger antiviral immunity but also enhance viral spread via this receptor.

Siglec-1 is a type I transmembrane lectin with an amino-terminal V-set domain that interacts with sialylated ligands, preferentially N-acetylneuraminic acid (Neu5Ac) in an α2–3 linkage [19,23]. Several enveloped viruses including human immunodeficiency virus (HIV)-1 [24,25,26] and Ebola virus (EBOV) [22,27,28,29] incorporate such sialylated ligands within their membranes as an integral part of the gangliosides that are dragged from the plasma membrane when viruses bud from infected cells. Although Siglec-1 affinity for sialylated ligands is in the micromolar range, high-avidity binding can be achieved upon clustering of thousands of gangliosides in the viral membrane with their receptors on the cellular membrane [19,30]. Moreover, as Siglec-1 contains 16 Ig-like C2-type extracellular domains that separate the ligand-binding site from the cell surface, it is available for interaction with external ligands and not bound in *cis* to cell-surface molecules, which is what usually happens with shorter Siglecs that are also expressed by DCs [19,23,31].

In vivo, the role of Siglec-1 during viral infection has been mostly studied in murine models, focusing on resident tissue macrophages that express this lectin and play key immunomodulatory functions. Siglec-1-expressing macrophages are located in the subcapsular sinus of the lymph nodes, and they protect mice against vesicular stomatitis virus (VSV) infection by containing incoming viruses. Viral sensing triggers cytokine release and promotes antigen presentation to B cells [32,33]. However, studies using different retroviruses to infect mice have shown that the protective function of these macrophages can be hijacked for efficient viral infection and dissemination within tissues. Indeed, robust infection of a particular retrovirus in lymphoid tissues and spleen requires Siglec-1-expressing macrophages [34]. The pathogenicity of the infecting retrovirus is key to tip the balance of these Siglec-1-expressing macrophages in favor of the protective immune function. The antiviral response dominates when the replicating virus has an expanded tropism [35], as it also happens in the case of the amphotropic VSV infection [32,33]. Under these pathogenic conditions, viral capture via Siglec-1 macrophages is necessary to elicit an effective antiviral CD8^+^ T cell response via antigen cross-presentation by DCs [35]. Overall, these murine studies explain how Siglec-1 can contain viral replication and induce antiviral immunity against highly pathogenic viruses, but also favor viral spread within tissues when retroviruses have a limited tropism. 

Yet, how these findings correlate with the pathogenesis of different Siglec-1-interacting human viruses, such as HIV-1 or EBOV, remains largely unexplored. HIV-1 is the causative agent of acquired immunodeficiency syndrome (AIDS), a pandemic that has affected more than 70 million people worldwide [36], while EBOV is responsible for the intermittent outbreaks that produce a filovirus-associated disease (FVD) with high fatality rates [37]. In this review, we discuss how Siglec-1 is induced on human DCs upon viral infection, to what degree that impacts different viral antigen presentation routes, and in which ways distant enveloped viruses have evolved to exploit Siglec-1 function as a dissemination strategy in distinct anatomical compartments. 

## 2. Viral Sensing and Immune Activation Triggers Siglec-1 Induction on DCs

**Figure 1:**
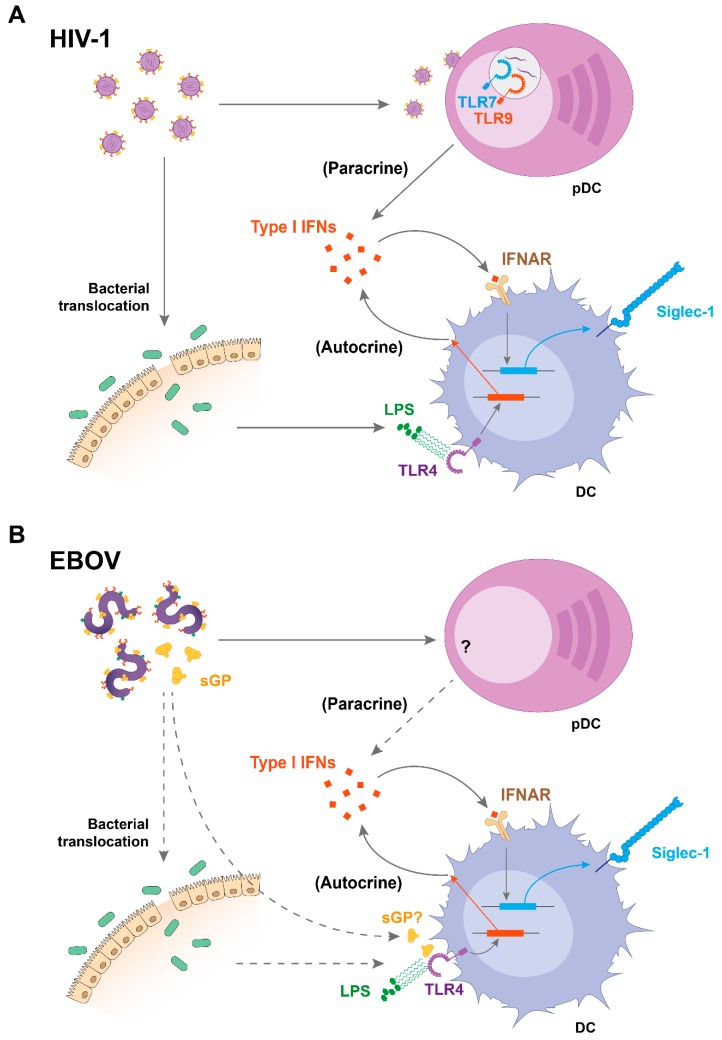
Mechanisms of Siglec-1 upregulation during human immunodeficiency virus (HIV)-1 and Ebola virus (EBOV) infections. (**A**) HIV-1 induces secretion of type I interferons (IFNs) by plasmacytoid DCs (pDCs) through Toll-like receptor (TLR) -7 and -9 sensing, which upregulates Siglec-1 on DCs in a paracrine manner. In addition, lipopolysaccharide (LPS) from bacterial translocation upregulates Siglec-1 on DCs via TLR4 sensing and autocrine type I IFN release. (**B**) During EBOV infection, type I IFNs might also play a central role in enhancing Siglec-1 expression on DCs, although this needs further investigation. pDCs may produce type I IFNs in response to EBOV infection in vivo, while bacterial translocation was suspected during a case of gram-negative septicemia in an EBOV-infected patient. In parallel, viral components such as secreted EBOV glycoprotein may induce activation of myeloid cells through TLR4 signaling, providing an alternative stimulus of autocrine type I IFNs during EBOV infection. While solid arrows indicate established mechanisms, dotted arrows suggest processes that require further investigation. IFNAR: IFNα/β receptor; sGP: secreted glycoprotein.

Siglec-1 is a receptor codified by an interferon-stimulated gene and is therefore potently upregulated on distinct human DCs, monocytes, and macrophages when these cells sense type I interferons (IFNs) such as IFNα [38,39,40]. Thus, infection with viruses such as HIV-1 or EBOV tightly upregulates Siglec-1 expression on APCs, as they directly trigger or indirectly promote the release of type I IFNs via immune activating factors (Figure 1). 

In the case of HIV-1 infection, IFNα levels are potently boosted during acute infection, and sustained—although to a lower extent—throughout the chronic stage, which is characterized by a persistent immune activation [41,42,43]. Several DC types have been identified as the sources of IFNα production during the course of HIV-1 infection, and therefore contribute to Siglec-1 induction. Plasmacytoid DCs (pDCs) are considered the most potent type I IFN producers in blood [44], and their capacity to secrete IFNα in response to HIV-1 sensing has been demonstrated in vitro [45,46,47,48,49] and in vivo [50,51,52], both during the acute and chronic phases of the disease [50,51,53]. Of note, pDC activation in response to HIV-1 sensing induces IFNα secretion through Toll-like receptor (TLR) -7 and -9 signaling [47,53] and maturation of bystander myeloid DCs [54], and this IFNα response is more potent on pDCs derived from females [55]. In turn, secretion of this cytokine can directly upregulate Siglec-1 expression on DCs [56] (Figure 1A). 

In addition to pDCs, myeloid DCs also secrete type I IFNs, although this release is mostly and indirectly triggered by immune activating signals present during the course of HIV-1 infection, which can induce the expression of IFN-stimulated genes on DCs in an autocrine manner [57,58,59,60]. One of those factors is bacterial lipopolysaccharide (LPS), which is increased in the plasma of HIV-1-infected individuals due to the bacterial translocation that takes place in the gut-associated lymphoid tissue as a consequence of the gut epithelial barrier disruption occurring early after HIV-1 infection [43,61,62] (Figure 1A). LPS induces Siglec-1 expression on DCs [63]. Moreover, plasma from HIV-1-infected individuals also stimulates Siglec-1 expression on DCs signaling via type I IFN receptor [40]. This explains why on circulating monocytes of HIV-1 infected individuals, Siglec-1 expression correlates in vivo with the levels of plasma viremia, and why these levels only diminish after introduction of combined antiretroviral treatment [40].

Moreover, both types of Siglec-1-inducing factors are also present throughout the course of EBOV infections (Figure 1B). Secretion of IFNα has been detected in humans and nonhuman primate models [64,65], especially in lethal cases [64], while asymptomatic EBOV infections are characterized by the absence of this cytokine [66]. Although in vitro pDCs exposed to EBOV do not secrete IFNα [67], activated pDCs have been found in EBOV-infected nonhuman primates, suggesting that these cells might produce IFNα in vivo [68] (Figure 1B). Aside from pDCs, myeloid cells could contribute to IFNα secretion during EBOV infection, as EBOV-like particles induce IFNα production by murine bone marrow-derived DCs through TLR signaling [69]. EBOV glycoprotein interaction with human monocyte-derived macrophages induced TLR4-dependent IFNα secretion by these cells [70]. Moreover, a cleaved and secreted form of EBOV glycoprotein signals through TLR4 [71], although IFNα secretion in response to these glycoproteins remains unexplored (Figure 1B). Noteworthy, LPS was also found in a case of EBOV infection complicated with septicemia, possibly due to bacterial translocation [72], which might account for indirect IFNα secretion during EBOV infection as described for HIV-1 (Figure 1B). 

Overall, the presence of type I IFNs throughout the course of these viral infections is well-established, although both HIV-1 and EBOV have evolved particular molecular mechanisms via viral antagonistic proteins that aid to evade cellular immune sensing [73,74,75,76]. Intriguingly, the protective role of type I IFN responses is controversial, since the apparent antiviral function during the earliest stages of infection may, in turn, fuel pathogenesis during the later stages of viral disease. That seems to be the case not only for HIV-1 [43], but also for EBOV [76], where clinical data collected during human outbreaks have indicated that elevated levels of circulating IFNα, as well as upregulation of type I IFN-inducible genes, correlates with fatal disease outcome [64,77,78,79]. Thus, HIV-1 and EBOV infections trigger an immune activation state that upregulates Siglec-1 expression on DCs, a situation that might favor early viral dissemination events in an otherwise antiviral environment [80].

## 3. The Role of Siglec-1 in DC Infection and Antigen Presentation 

**Figure 2:**
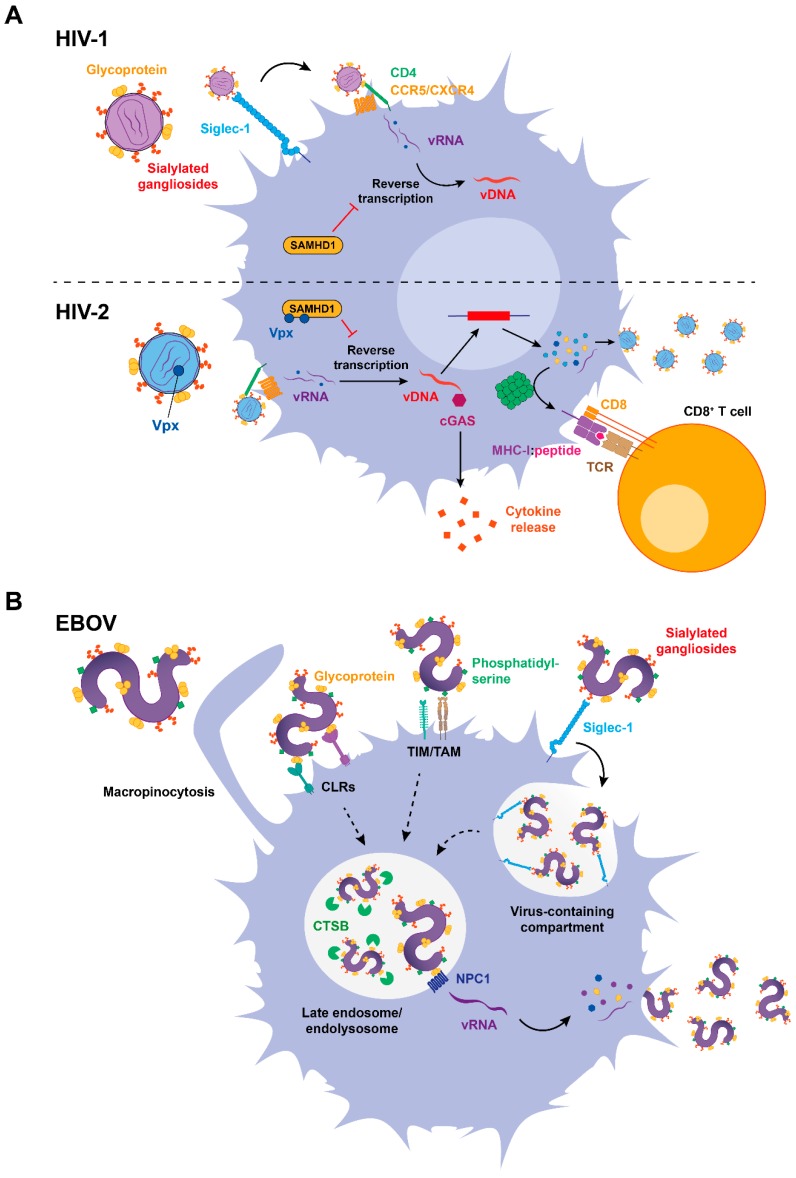
Role of Siglec-1 in DC infection and antigen presentation. (**A**) Top: Siglec-1 facilitates HIV-1 infection of antigen presenting cells (APCs). In most DC subtypes, however, the restriction factor SAMHD1 inhibits reverse transcription, thus precluding immune sensing and the synthesis of viral antigens. Bottom: Conversely, HIV-2 encodes Vpx, which counteracts SAMHD1 activity allowing reverse transcription of the viral genome, that can be sensed via cGAs and trigger cytokine release. Newly synthesized proteins lead to the production of viral particles, but also to proteosomal cleavage and viral antigen presentation to CD8^+^ T cells via major histocompatibility complex class I (MHC)-I. (**B**) EBOV employs different receptors to attach to target cells, including CLRs, TIM/TAM receptors, and Siglec-1. Upon internalization, EBOV is directed to late endosomes and interacts with NPC1 receptor after being processed by cell cathepsins. In the cytoplasm, EBOV replicates producing new viral proteins and synthesized virions bud. While solid arrows indicate established mechanisms, dotted arrows suggest processes that require further investigation. vRNA: viral RNA; vDNA: viral DNA; SAMHD1: sterile alpha motif (SAM) domain- and histidine-aspartate (HD) domain-containing protein 1; cGAS: cyclic GMP-AMP synthase; TCR: T cell receptor; CLRs: C-type lectin receptors; TIM: T cell immunoglobulin and mucin domain receptor; TAM: Tyro-Axl-Mer receptor; CTSB: cathepsin B; NPC1: Nieman–Pick C1 receptor.

Viral sensing enhances Siglec-1 expression on APCs, and this facilitates HIV-1 infection of DCs and other myeloid cells (Figure 2A, top), such as macrophages [81]. Although DCs are generally resistant to HIV-1 infection, a recent study showed that Siglec-1 mediated HIV-1 productive infection of a population of human DC precursors known as pre-DCs [82]. Even though all DCs express the HIV-1-interacting cellular receptor CD4 and the viral coreceptors [83,84], being therefore susceptible to viral infection in vitro [85,86,87], infectivity was less prominent than in activated CD4^+^ T cells [88,89,90,91]. Importantly, the activation process that enhances Siglec-1 expression on DCs further restricts viral infection, as mature DCs are 10-fold to 100-fold less susceptible to HIV-1 than immature DCs [9,88,92,93,94]. Infection of DCs with HIV-1 also appears to be uncommon in vivo, although it has been reported for both cutaneous and mucosal DCs, including vaginal epithelial DCs [91,95,96]. Yet, the role of Siglec-1 in promoting HIV-1 infection of these DCs remains largely unexplored.

The de-phosphorylated form of the host factor sterile alpha motif histidine-aspartate domain-containing protein 1 (SAMHD1) is the most potent restriction factor that limits HIV-1 infection in DCs [97] (Figure 2A, top). However, if this lack of infectivity is actually beneficial for an immune control remains controversial, as the absence of viral replication impairs viral sensing and limits presentation of HIV-1-specific antigens to prime adaptive immune responses [98,99,100]. In contrast to HIV-1, HIV-2 naturally replicates on DCs [98], which depends on the counteraction of SAMHD1 by the viral antagonist Vpx [91,92,93,94,95,96,97,98,99,100,101] (Figure 2A, bottom). HIV-2 genome replication in infected DCs is detected by the innate cytosolic DNA sensor cyclic guanosine-adenosine monophosphate synthase (cGAS), which triggers immune responses upon DNA sensing [98,99] (Figure 2A, bottom). In contrast, HIV-1 restriction by SAMHD1 prevents viral DNA (vDNA) retrotranscription in the cytoplasm, impairing the induction of antiviral type I IFN responses [98] (Figure 2A, top). Thus, viral replication on DCs can provide an additional source of viral components to be detected via immune sensors or presented to T cells [98,102] (Figure 2A, bottom), but also compromise cell viability and release inflammatory factors that fuel viral pathogenesis, as previously described for sustained or chronic type I IFN responses. 

While HIV-1 replication on DCs remains hard to identify *in vivo*, it has been known for more than a decade that DCs are among the first target cells encountering EBOV [11]. DCs are highly susceptible to EBOV infection [11,103], and this is a complex process that involves several host factors whose function is still being identified [104]. Indeed, Siglec-1 expressed on activated DCs has recently emerged as a new host factor implicated in EBOV attachment, a mechanism that facilitates subsequent cytoplasmic viral entry [22] (Figure 2B). 

Initial EBOV attachment to the DC surface is mediated by several receptors that recognize different elements on the viral membrane and often have a redundant activity [105]. C-type lectin receptors (CLRs) such as the dendritic cell-specific intercellular adhesion molecule-3-grabbing non-integrin (DC-SIGN) and the liver/lymph node sinusoidal endothelial C-type lectin (LSECtin) mediate viral attachment through binding to viral glycoproteins [106,107], while receptors of the TIM/TAM families (comprising the T cell immunoglobulin and mucin domain receptor along with Tyro-Axl-Mer receptors) recognize phosphatidylserine lipids present on the viral envelope [108] (Figure 2B). EBOV incorporates sialylated gangliosides on their membrane [27], and we have recently shown that these viruses are effectively recognized by the Siglec-1 receptor [22] (Figure 2B).

Siglec-1 recognition of sialylated gangliosides on EBOV modulates the binding, uptake, and trafficking of filoviral particles into a sac-like virus-containing compartment (VCC) continuous with the plasma membrane (Figure 2B). Viruses stored in this compartment can be redirected into the classical endosomal pathway and facilitate viral entry into the cytoplasm [22]. Indeed, EBOV macropinocytosis allows trafficking into late endosomes, where cleavage of viral glycoproteins by cathepsin B (CTSB) facilitates the interaction with the endosomal receptor Niemann–Pick C1 [109,110,111] (NCP1) that triggers cytoplasmic viral entry [112,113,114] (Figure 2B). Thus, Siglec-1-mediated attachment facilitates viral access to the cell cytoplasm [22]. 

While Siglec-1 contributes to EBOV entry into DCs, filoviral replication within these cells compromises immune function and prevents adaptive immune responses by limiting cytokine secretion, downregulating the expression of major histocompatibility complex (MHC) and costimulatory molecules and also by reducing the ability of DCs to stimulate T cell proliferation [103,115,116,117]. These results suggest that EBOV suppression of DC function prevents initiation of adaptive immune responses and facilitates uncontrolled systemic virus replication [116,117] through a mechanism that is enhanced by Siglec-1 activity [22]. However, clinical data gathered during the West African 2014–2016 outbreak showed strong and sustained T cell activation [118], challenging the *in vivo* relevance of this viral DC-escape mechanism [76].

Overall, both HIV-1 and EBOV can exploit Siglec-1 activity to boost DC infectivity, although EBOV replication is more prominent in these cells. Yet, viral infection poses a difficult balance for APCs. On the one hand, infectivity triggers antiviral immunity via viral sensing and antigen presentation, but on the other hand, it also promotes cell death and suppression of immune responses through the activity of particular viral antagonist proteins. Recently, it has been suggested that this apparent paradox is overcome by a division of labor between distinct DC subsets [119]. There is therefore a dissociation between viral infection and antigen presentation, which occurs in distinct DC subpopulations. By these means, susceptible infected DCs transfer viral antigens to resistant DCs, which remain competent to launch adaptive immune responses against viral infections [119]. 

## 4. Siglec-1 Captures Antigen-Containing Extracellular Vesicles, but This Mechanism also Promotes Viral *Trans*-Infection

**Figure 3:**
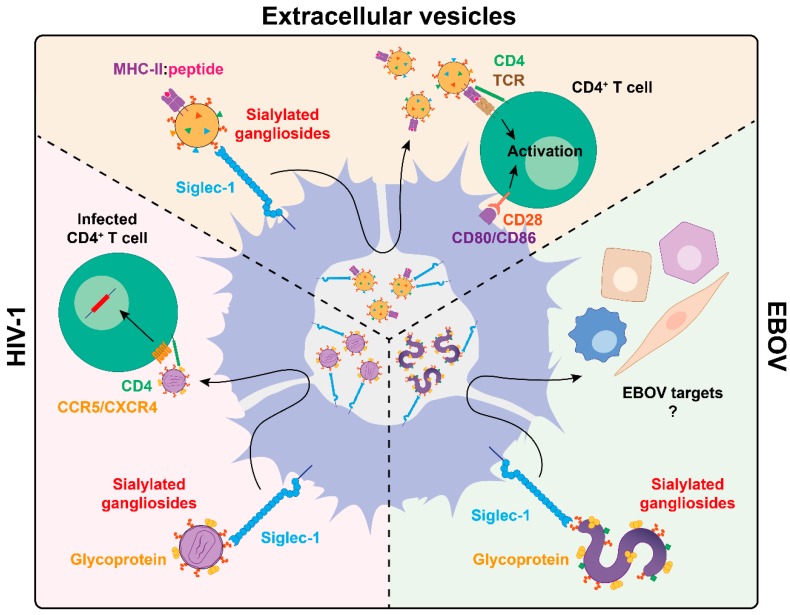
Viral subversion of Siglec-1-mediated extracellular vesicle dissemination. DCs capture extracellular vesicles bearing MHC–peptide complexes or distinct enveloped viruses such as HIV-1 or EBOV through Siglec-1 recognition of sialylated gangliosides and are then trafficked and stored within a sac-like compartment. While captured extracellular vesicles exit this compartment to present antigens to T cells via immune synapse formation complemented by the co-stimulatory signals provided by the activated DC, the exit of HIV-1 leads to the *trans*-infection of CD4^+^ T cells. In the case of EBOV, viral dissemination to other target cells needs further investigation. MHC-II: major histocompatibility complex class II; TCR: T cell receptor.

While several pathways allow for antigen transfer between DCs, secretion of extracellular vesicles bearing particular antigens is among the most effective ones. Although the functional paradigm of DC biology states that the particular APC that interacts with incoming viruses in the mucosa would be the one processing these viruses and then traveling to the lymphoid tissue, these cells may not always be the only ones presenting the captured antigens. Rather, these pathogen-interacting DCs may transfer captured antigens to other APCs by several mechanisms, including secretion of extracellular vesicles bearing antigen-loaded fragments, which can even be already processed and presented in MHC molecules (Figure 3, top). By these means, the number of DCs bearing viral-specific antigens can be increased very quickly upon infection, thus amplifying the initiation of primary adaptive immune responses [120,121,122]. Importantly, to induce naïve T cell stimulation *in vitro*, these extracellular vesicles require a competent activated DC to deliver the co-stimulatory signals to T cells [121] (Figure 3, top). Thus, antigen-containing extracellular vesicles do not overcome the need for a competent APC to activate naïve T cells.

Among the distinct cellular receptors expressed by DCs, Siglec-1 is key to capture secreted extracellular vesicles through the same mechanism hijacked by enveloped viruses [123] (Figure 3). Siglec-1 interacts with extracellular vesicles via recognition of sialylated gangliosides packaged on the vesicle membrane [124], which assemble and bud from cellular membranes as viruses do [125,126]. This result has been confirmed not only in vitro [63,127] with extracellular vesicles derived from cell lines or primary cells but also in murine models where Siglec-1 expressed on lymphoid tissues was required to trap extracellular vesicles *in vivo* [128].

Upon capture of extracellular vesicles on activated DCs via Siglec-1, these vesicles are trafficked along with the receptor towards a sac-like compartment invagination that is continuous with the plasma membrane and allows for extracellular vesicle retention [63,123] (Figure 3, top). The Siglec-1 positive compartment formed within activated DCs may serve as an antigen depot, controlling and sustaining adaptive immunity even if the source of antigen is not directly in contact with the APC, that still can trigger antigen-specific immune responses. These antigens could maintain immunity for prolonged periods, as it happens when DCs control endosomal acidification to preserve antigen cross-presentation over time [129]. Although mature or activated DCs markedly downregulate their macropinocytic capacity, these cells are still able to capture, process, and present antigens internalized via endocytic receptors [130], and that may also be the case for Siglec-1 via extracellular vesicle trapping. Moreover, as DCs continue to capture and present antigens after maturation *in vivo* [131], they could also initiate responses to newly encountered antigens during the course of viral infections, a process that would be boosted by Siglec-1 expression.

The fate of trapped extracellular vesicles on DCs is diverse, as they provide a source not only for antigen cross-presentation to CD8^+^ T cells, but also to stimulate antigen-specific naïve CD4^+^ T cell responses *in vivo* [120,121]. CD4^+^ T cell stimulation can take place either by reprocessing the antigens contained in the captured extracellular vesicles or by the direct presentation of previously processed functional epitope–MHC complexes exposed in the vesicle surface [120,121]. Direct extracellular vesicle antigen presentation in the absence of lytic degradation within DCs was initially described using DC populations devoid of particular MHC-II molecules, that were still able to activate CD4^+^ T cells because the necessary MHC-II molecules were already presenting the antigen on the extracellular vesicles trapped by those DCs [121]. Thus, extracellular vesicles displaying previously processed functional epitope–MHC complexes on their surface can be recognized, retained, and directly transferred from DCs to antigen-specific CD4^+^ T cells [121] (Figure 3, top). In turn, Siglec-1 upregulation on activated DCs, which are competent APCs, could boost extracellular vesicle uptake and magnify antiviral immune responses.

Intriguingly, HIV-1 and other retroviruses exploit this antigen dissemination pathway usually engaged by extracellular vesicles to reach CD4^+^ T cells [123], which are the main cellular targets of this particular retrovirus (Figure 3, bottom left). Siglec-1 directs captured HIV-1 particles to the same VCC where Ebola viral particles are retained [22], that is in addition the same compartment where extracellular vesicles are trapped in activated DCs [63,123] (Figure 3, bottom left). However, in the case of HIV-1 recognition, viral entry via Siglec-1 does not lead to the productive infection of DCs as it happens with EBOV, but favors the transfer of trapped viruses to bystander CD4^+^ T cells. Thus, trapped viruses are efficiently transmitted across infectious synapses to susceptible lymphocytes [9,132,133] (Figure 3). This mechanism of viral transmission is known as *trans*-infection [134] and is mediated by Siglec-1 on activated monocyte-derived DCs, monocytes, blood conventional DCs, pre-DCs, and primary myeloid cells isolated from lymphoid and cervical tissues [38,39,40,56,63,82].

Filoviral *trans*-infection from DCs to CD4^+^ T cells is improbable as lymphocytes are largely resistant to EBOV infection [135]. Nonetheless, filoviruses display a broad cell tropism, infecting hepatocytes, adrenal cortical cells, and endothelial cells, among other cellular targets [136,137,138,139]. Thus, aside from lymphocytes, other cellular targets could be transinfected (Figure 3, bottom right), as it was previously shown for a human cell line binding EBOV that transinfected HeLa cells [140]. However, further research will be required to determine in which anatomical context DCs trapping EBOV via Siglec-1 could transfer that infectivity to susceptible cellular targets *in vivo*. 

Overall, Siglec-1 retention of distinct viruses on extracellular vesicle-containing compartments highlight how these cells might act as “Trojan Horses”, capturing filoviruses or retroviruses in the peripheral mucosae and carrying them to secondary lymphoid tissues, where viruses can be effectively transmitted to target cells and contribute to the systemic spread of infection [9,10,11,94,141]. 

## 5. Siglec-1 Expression on Different Anatomical Compartments and Viral Dissemination Routes

**Figure 4:**
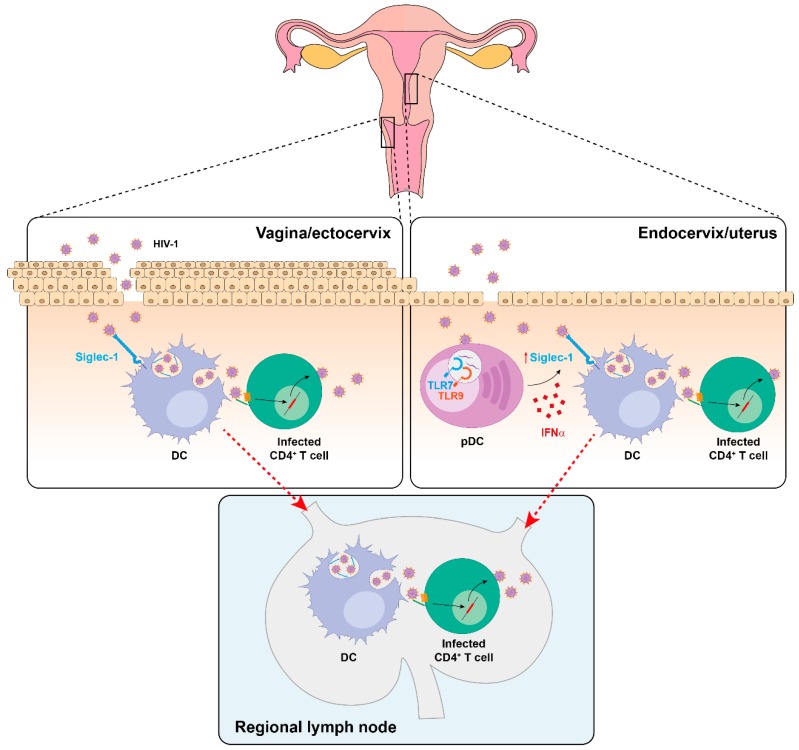
Proposed mechanism of HIV-1 dissemination from the female reproductive tract. In the vaginal/ectocervical mucosa, basal Siglec-1^+^ DCs may mediate local HIV-1 *trans*-infection to target CD4^+^ T cells. At the endocervical mucosa, lower Siglec-1 expression is boosted in response to IFNα released by recruited pDCs sensing HIV-1. Siglec-1^+^ DCs may also contribute to systemic HIV-1 spread due to their ability to migrate to secondary lymphoid tissues, where CD4^+^ T cells accumulate. While a similar mechanism could be exploited by EBOV, further work needs to address this possibility.

Recently, we have identified the presence of Siglec-1-expressing cells in cervical mucosa. These DCs are capable of capturing HIV-1 and mediate viral transmission to target CD4^+^ cells via *trans*-infection, even in a basal state where no apparent activation is detected [56] (Figure 4). Indeed, DCs directly isolated from human ectocervix displayed a basal Siglec-1 expression that was sufficient to mediate viral transfer. However, at the endocervix, where the expression of Siglec-1 is lower than at the ectocervix, this capacity was enhanced upon IFNα stimulation (Figure 4). 

HIV-1 is mostly acquired by sexual transmission, and in the cervical mucosa, there are two major sources of antiviral type-I IFN responses after retroviral infection: resident myeloid cells [142] and pDCs, which are the most potent producers of IFNα [44] and are soon recruited to the cervix [143] (Figure 4). Although increased antiviral IFNα secretion could limit initial viral infection, it could promote viral capture on cervical myeloid cells via Siglec-1 induction as well. Of note, in the cervical biopsy of a viremic HIV-1^+^ patient, Siglec-1^+^ cells harbored HIV-1-containing compartments, demonstrating that in vivo, these cells can trap viruses [56]. Interestingly, similar VCC-like structures have been detected in urethral macrophages of HIV-1-infected individuals under suppressive combination antiretroviral therapy [144], but if Siglec-1 is implicated in the formation of these particular structures remains to be determined. 

Siglec-1 allows transferring viruses to bystander CD4^+^ T cells in the mucosa, but also the systemic viral dissemination upon DC migration to lymphoid tissues (Figure 4). Indeed, DCs bearing retroviruses are found in the draining lymph nodes of distinct animal models as soon as 24 h after vaginal challenge [12,145,146,147] and these findings originally led to formulation of the Trojan Horse hypothesis, which states that DCs can serve as vehicles transporting the virus from the entry sites to distant tissues [9,10].

Sexual transmission is not considered a major route of EBOV infection. However, a case of sexually transmitted EBOV has been well documented [148]. Moreover, a recent mathematical model is consistent with a significant contribution of sexual EBOV transmission during the 2014–2016 outbreak in West Africa [149]. Importantly, infectious viral particles are found in semen of EBOV convalescent individuals several months following symptoms onset [150,151,152,153], and seminal fluid amyloids may enhance EBOV infection [154]. As the cytokine TGF-β1 is abundant in semen, and it also upregulates Siglec-1 expression on DCs [155], the role of this receptor should be further assessed in the context of EBOV sexual transmission.

Moreover, DCs are early and sustained targets of EBOV that can disseminate infection from the portals of viral entry to the regional lymph nodes, spleen, and liver [11]. Since Siglec-1 is expressed in all these EBOV replicating-tissues [23,156], this receptor could also boost systemic viral spread as previously suggested for HIV-1 [11,13,139,157,158]. Collectively, these data indicate that Siglec-1 on DCs may not only participate in viral transmission at the mucosa, but also promote systemic viral dissemination to secondary lymphoid tissues.

## 6. Future Perspectives

We hypothesize that the outcome of early interactions between DCs and enveloped viruses may be key to mount effective antiviral responses, but these early encounters also foster viral dissemination to distant tissues. The emerging roles of Siglec-1 receptor on DCs clearly exemplify how the immune-mediated activity of this lectin can be effectively hijacked by unrelated viruses such as HIV-1 or EBOV. Future work should address if other enveloped viruses causing relevant human infectious diseases may contain sialylated gangliosides on their membranes and be recognized by Siglec-1, as it has been already shown for the henipavirus [159]. Moreover, the precise contribution of Siglec-1 to viral immune containment or to pathogenic viral dissemination should be carefully evaluated, as understanding both mechanisms may provide novel avenues to combat infectious agents. 

The identification of individuals which naturally lack Siglec-1 expression due to the presence of an early stop codon in the *SIGLEC1* gene in homozygosis [160] indicates that the role of this protein is not essential and that it may be therefore a safe therapeutic target. Developing such pharmacological agents to block Siglec-1 interaction with viruses could pave the way to ameliorate viral systemic dissemination. Moreover, exploiting the immune-surveillance function of Siglec-1 receptor to induce antiviral immune responses may also prove valuable. In turn, dissecting how distinct viruses exploit common molecular pathways will advance future antiviral strategies to generate broad spectrum treatments.
